# Proactive reward in conflict tasks: Does it only enhance general performance or also modulate conflict effects?

**DOI:** 10.3758/s13414-024-02896-5

**Published:** 2024-06-24

**Authors:** Linda C. Bräutigam, Hartmut Leuthold, Ian G. Mackenzie, Victor Mittelstädt

**Affiliations:** Department of Psychology, University of Tübingen, Schleichstrasse 4, 72076 Tübingen, Germany

**Keywords:** Proactive reward, Conflict tasks, Congruency effects, Delta plots, Cognitive control

## Abstract

In the present study, we investigated the influence of performance-contingent reward prospects on task performance across three visual conflict tasks with manual responses (Experiments 1 & 2: Simon and Stroop tasks; Experiment 3: Simon and Eriksen flanker task) using block-wise (Experiment 1) and trial-wise (Experiments 2 & 3) manipulations to signal the possibility of reward. Across all experiments, task performance (in reaction time and/or error rates) generally improved in reward compared with no-reward conditions in each conflict task. However, there was, if any, little evidence that the reward manipulation modulated the size of the mean conflict effects, and there was also no evidence for conflict-specific effects of reward when controlling for time-varying fluctuations in conflict processing via distributional analyses (delta plots). Thus, the results provide no evidence for conflict-specific accounts and instead favor performance-general accounts, where reward anticipation leads to overall performance improvements without affecting conflict effects. We discuss possible implications for how proactive control might modulate the interplay between target- and distractor-processing in conflict tasks.

## Introduction

Goal-directed behavior requires cognitive control, the ability to flexibly adjust information processing (sensory, central, and motor) according to internal goals and situational demands (e.g., Braem & Egner, 2018; Braver, 2012; Verbruggen et al., 2014). In real-world situations, people are often informed that their behavior can produce additional positive or negative outcomes (i.e., reward or punishment) depending on performance. Thus, the role of rewards in motivating and influencing goal-directed behavior has become a central topic in cognitive psychology and neuroscience research (e.g., Chen et al., 2021; Frömer et al., 2021; Hefer & Dreisbach, 2017; Krebs & Woldorff, 2017; Mittelstädt et al., 2022). Previous research has shown that anticipating performance-contingent rewards can improve cognitive control as reflected in enhanced task performance relative to a condition without reward (e.g., Bundt et al., 2016, 2021; Yamaguchi & Nishimura, 2019). However, it remains unclear whether reward anticipation can have additional effects on behavior when making decisions in situations with potentially distracting (conflicting) information. Specifically, previous studies have reported mixed findings regarding whether reward manipulation in conflict tasks improves performance in general or also influences conflict effects (e.g., Padmala & Pessoa, 2011; Soutschek et al., 2015). In the present study, we aim to provide further insights about this issue by rigorously testing the influence of proactive (i.e., preparatory processes in advance of trials; cf. Braver, 2012) reward manipulations across multiple prominent conflict tasks—namely, the Simon (Simon, 1990), Stroop (Stroop, 1935), and Eriksen flanker task (Eriksen & Eriksen, 1974).

In conflict tasks, participants are required to respond to task-relevant (target) information (e.g., color) while ignoring task-irrelevant distracting information (distractors). The specific type of distractor thereby varies across the three most prominent conflict tasks: the location of the target in the Simon task, the flankers surrounding the target in the Eriksen flanker task, and the ink color of the written target word in the Stroop task (e.g., Kreutzfeldt et al., 2016; Servant & Logan, 2019; Wühr & Heuer, 2018). In other words, conflict is caused by different types of distractor information—namely, position (Simon), identity (Flanker), and semantic (Stroop) information. However, all these conflict tasks have in common that task performance (reaction time [RT] and/or error rates [ER]) is influenced by the presence of distractors, as reflected in better performance when the target and distractor indicate the same (congruent) compared with different (incongruent) responses. Theoretical explanations of these so-called conflict effects (CEs) posit that activations produced by both the distractor-based and target-based information superimpose when selecting a response, resulting in facilitation or impairment of task performance (e.g., Botvinick et al., 2001; Coles et al., 1985; De Jong et al., 1994; Eimer et al., 1995; Hübner et al., 2010; Miller & Schwarz, 2021; Mittelstädt & Miller, 2020; Ridderinkhof et al., 1995; Stürmer et al., 2002; Ulrich et al., 2015; Wühr & Heuer, 2018). Thus, it seems plausible to integrate conflict processing involving different distractors into unified processing frameworks, thereby assuming shared cognitive control mechanisms across these tasks (e.g., O’Leary & Barber, 1993; Ulrich et al., 2015).

Several empirical studies indeed support the idea that (at least partially) distractor-general control processes are involved in dealing with various conflict effects (e.g., Mittelstädt et al., 2023a; Peterson et al., 2002; Treccani et al., 2009). For example, both Flanker and Simon effects are larger when the relevance of the distractor increases, and additional distributional (delta plots) and model-based analyses suggest that this increase arises from similar control adjustments (Mittelstädt et al., 2023a). Furthermore, both location-based and flanker-based distractor activation are assumed to produce conflicts at the same processing stage (i.e., response selection; cf. Treccani et al., 2009). However, it is often necessary to additionally propose the operation of distractor-specific control processes across various conflict tasks (e.g., Egner et al., 2007; Kornblum et al., 1990) to account for certain empirical findings (e.g., Mackenzie et al., 2022; Mittelstädt et al., 2023a; Scerrati et al., 2017). For example, in a combined Stroop–Simon paradigm, the Stroop- and Simon-like effects were additive, suggesting that conflicts occur at different processing stages (i.e., perception in the Stroop and response selection in the Simon task; cf. Scerrati et al., 2017). As will be discussed next, distractor-specific control processes also seem relevant to consider regarding the influence of reward manipulations on congruency effects.

Several studies have investigated the influence of reward manipulations on processing in conflict tasks (e.g., Botvinick et al., 2001; Braem et al., 2012; Carsten et al., 2019; Kang et al., 2018; Krebs et al., 2010; Prével et al., 2021; Veling & Aarts, 2010). However, most relevant for the present study are situations where participants are informed before a task is performed that they can receive reward based on their performance (i.e., performance-contingent proactive reward manipulation). Surprisingly, to our knowledge, only a few studies have directly applied such a manipulation—that is, using a cue in advance of each trial using a cue in advance of each trial (e.g., Bundt et al., 2016, 2021; Padmala & Pessoa, 2011; Veling & Aarts, 2010; Yamaguchi & Nishimura, 2019) or between different blocks (e.g., Kang et al., 2018; Soutschek et al., 2015) to signal reward availability for good performance (see Table 1). These studies have consistently shown that the potential for reward enhances task performance, presumably due to increased motivation and/or alertness that leads to proactive biases in processing the target and/or initiating a response (e.g., Bowers et al., 2021; Braver, 2012). However, partially conflicting results have been observed regarding the effects of distracting information on behavior with reward manipulation. While some studies have observed no effect of reward on conflict effects (Bundt et al., 2016), others have reported reduced (Padmala & Pessoa, 2011; Soutschek et al., 2015; Yamaguchi & Nishimura, 2019) and increased (Bundt et al., 2021) conflict effects in reward compared with no-reward conditions.

**Table 1:** Overview of previous studies investigating the influence of proactive reward manipulations in conflict tasks

Study	Task	Reward manipulation	Overall reward effect		Reward-specific congruency effects	
			Reaction time	Error rate	No-reward	Reward
Bundt et al. (2016)	Simon task (manual responses to visual targets)	Trial-wise & fixed RT threshold (700 ms)	15 ms*	~ 0.6%	~25ms;	~25 ms;
					~ 2%	~ 1.8%
Bundt et al. (2021)	Stroop task (manual responses to visual color)	Trial-wise & fixed RT threshold (700 ms)	27 ms*	1.3%*	24ms*;	37 ms*;
					~ 2%	~ 1.7%
Padmala and Pessoa (2011)	Compound scene-plus-word stimuli (manual responses to visual targets)	Trial-wise & RT threshold (800 ms)	~40 ms*	~4% *	~65 ms*;	~ 40 ms*;
					~ 8%*	~ 4%*
Soutschek et al. (2015)	Stroop-like picture-word task (manual responses to visual target)	Block-wise & RT threshold (adaptive)	66 ms*	−0,60%*	34 ms*;	26 ms*;
					4.8%	4.95%
Yamaguchi and Nishimura (2019), Exp. 1	Flanker (manual responses to visual target color)	Trial-wise & fixed RT threshold 1.5 s (Trial threshold)	13 ms*	0.36%	28 ms*;	15 ms*;
					1.24%	0.53%
van den Berg et al. (2014)	Stroop Task (manual responses to visual color)	Trial-wise & RT threshold (adaptive)	~30ms*	2.0%*	~65 ms	~ 65 ms
					n.a.	n.a.
Kang et al. (2018)	Crossmodal conflict-task paradigm (manual responses to either auditory or visual living vs. nonliving objects)	Block-wise & RT threshold (adaptive)	65ms*	−1.1%*	16 ms*	~5 ms*
					~0.3%	~−0.5%

Overall reward effects in reaction times (RTs) and error rates (ERs) reflect differences in no-reward minus reward conditions, except for the study by van den Berg et al. (2014, loss versus gain). The asterisk (*) indicates that the respective main effect was significant (*p* < .05). Reward-specific conflict effects illustrate the difference in RTs and ERs in incongruent minus congruent conditions separately for the no-reward and reward conditions. Here, the asterisk (*) indicates that the interaction between reward and congruency was significant (*p* < .05). Note that when the specific values were not directly provided in the text, we estimated the values based on visual inspection of the corresponding figures or by collapsing across conditions. Note that in the study by van den Berg et al. error rates as a function of congruency and reward were not available (n.a,)

On a theoretical level, it is possible to consider both performance-general accounts where reward anticipation leads to overall performance improvements, and conflict-specific accounts, where such anticipation can also affect conflict effects. In general, proactive control describes preparatory processing adjustments before the presentation of targets and distractors (e.g., Braver, 2012). According to performance-general accounts, then, for example, the reward manipulation may only bias processes that are not directly involved in the superimposition of activation (e.g., early sensory and/or late motoric processes). According to conflict-specific accounts, reduced conflict effects in reward compared with no-reward conditions may be observed if target enhancement and/or distractor suppression at the activation-superimposition stage leads to increases in the signal (target) to noise (distractor) ratio. Furthermore, the reversed pattern might be observed if increased sensitivity to distractors under reward reduces the signal-to-noise ratio at the superimposition stage, leading to increased conflict effects.

However, before further elaborating on the specific underlying processes, it seems useful to distinguish between these behavioral accounts and identify whether a performance-contingent proactive reward manipulation produces similar effects across different conflict tasks. As mentioned earlier, this is particularly important since there are generally only a few studies applying this type of manipulation in conflict tasks. Moreover, as described in more detail next, the potential for generalization across different conflict tasks is a priori questionable based on the currently available empirical evidence and theoretical reasons (e.g., distractor-specific control processes).

Specifically, the findings are mixed, but Table 1 indicates that the majority of studies supporting conflict-specific accounts when using a proactive performance-contingent reward manipulation have used versions of a Stroop task (e.g., Bundt et al., 2021; Soutschek et al., 2015), whereas performance-general accounts are only supported from a Simon task study (Bundt et al., 2016). Furthermore, conflict-specific accounts also receive empirical support from previous studies employing a performance-contingent manipulation where rewards were linked to specific target features (e.g., Krebs et al., 2010; Wang et al., 2019) or conflict (e.g., Chen et al., 2021; Mittelstädt et al., 2023b; Prével et al., 2021).^1^ For example, in the Stroop task, Krebs et al. (2010) observed reduced conflict effects when rewards were linked to specific target features (i.e., colors) compared with no-reward target features, while Wang et al. (2019) observed the exact opposite pattern in the Simon task. Additionally, in the Simon task, reduced conflict effects were found when reward was associated with incongruent compared with congruent trials (Chen et al., 2021; Mittelstädt, Ulrich et al., 2023b), whereas the Stroop task generally showed little modulation of conflict effects with this type of reactive performance-contingent reward manipulation (Mittelstädt et al., 2023b, Prével et al., 2021).

Hence, these findings may suggest that (at least some) control processes in the Stroop task, in comparison with the Simon task, are differentially sensitive to reward manipulations. For example, it could be particularly critical to consider the processing stages at which conflict effects primarily occur in these tasks. In the Stroop tasks, distractors primarily elicit perceptual-related informational and higher-level task conflict, whereas the Simon task mainly generates motor-related informational conflict (cf. Goldfarb & Henik, 2007; Mittelstädt et al., 2023b; Rey-Mermet et al., 2019; Scerrati et al., 2017; Steinhauser & Hübner, 2009). Now, consider, for example, that a proactive reward manipulation might selectively enhance perceptual target processing without affecting later processing stages. While performance should generally improve in both tasks, under this assumption, only the Stroop effect, but not the Simon effect, will be reduced.

Motivated by this theoretical rationale and the generally limited and partially inconclusive empirical evidence, the major goal of the present study was to shed more light on the potential effects of proactive performance-contingent reward manipulations in terms of conflict-specific and performance-general accounts across various conflict tasks while controlling for other methodological aspects as much as possible (e.g., using the same target dimensions, number of trials per conflict task as well as instructions). For this purpose, we focused on the Simon and Stroop task in Experiment 1 and 2. To foreshadow, while performance was generally improved with reward compared with without, the results provided no evidence for conflict-specific accounts. Subsequently, we decided to include the Eriksen flanker task in Experiment 3. While the control processes in this task may share partial similarities with those in the Simon (e.g., Treccani et al., 2009) and Stroop tasks (e.g., Verbruggen et al., 2014), there are likely also flanker-specific control processes such as involving the biasing of spatial attention (cf. Mattler, 2005; White et al., 2011). Hence, it remained unclear whether a performance-general account would apply to this conflict task, even when taking into account the findings of a previous study: Specifically, Yamaguchi and Nishimura (2019) observed reduced flanker effects in a performance contingent-reward compared with a no-reward condition (Experiment 1). However, the authors noted that this could reflect a timing difference between conditions, since a reward cue, but not a no-reward cue, was presented in advance of each trial. Overall, then, the present study allowed us to gain insights into the outcomes of proactive performance-contingent reward manipulations (conflict specific vs. performance general) while considering potential distractor-specific versus distractor-general control mechanisms. Specifically, it provided a test as to whether changes in proactive control based on the reward manipulation only bias processing with different distractors in a way to generally improve performance or also influence the size of conflict effects.

When investigating the effects of reward and conflict processing, it is useful to complement traditional mean-based analysis with distributional analysis, specifically delta plots (e.g., De Jong et al., 1994; Gade et al., 2020; Heuer et al., 2023; Hübner & Töbel, 2019; Kelber, Gierlich et al., 2023a; Luo & Proctor, 2020). Delta plots show the conflict effect across the RT distributions. Typically, the size of the conflict effect is not independent of response speed. Instead, primarily negative-going delta plots are found in the visual Simon task, indicating larger conflict effects for faster responses (e.g., Ellinghaus et al., 2018; Hazeltine et al., 2011; Pratte et al., 2010), whereas primarily positive-going delta plots are found in the Stroop and Eriksen flanker tasks, indicating smaller conflict effects for faster responses (e.g., Mackenzie et al., 2022; Pratte, 2021; Ridderinkhof et al., 2004; Ulrich et al., 2015). Such different slopes in the present study would demonstrate the presence of conflict task-specific processes, such as differences in the speed of distractor processing and/or in the locus of superimposition. More important, because the reward manipulation influences the overall response speed, the corresponding conflict effects on mean RT may be explainable solely by the unfolding of distractor-based activation across time (Mittelstädt & Miller, 2018, 2020; Mittelstädt et al., 2022) or not (Kelber et al., 2023b; Mittelstädt & Miller, 2020). Furthermore, some studies suggest that conflict-specific control processes can affect the slopes of delta plots (e.g., faster distractor suppression leads to steeper delta plot slopes, see Hübner & Töbel, 2019; Ridderinkhof et al., 2004; Ridderinkhof, 2002). Therefore, the present delta plot analyses will help to control for time-varying distractor-based activation when interpreting effects on mean RT (by comparing the size of condition-specific conflict effects at the same mean RT) and to detect potential effects on the timing of cognitive control (by comparing the condition-specific delta plot slopes).

## Experiment 1

In the first preregistered Experiment, we investigated how a block-wise proactive reward manipulation influenced performance (including the size of conflict effects) in the Simon and Stroop tasks. In addition to general performance improvements with reward compared with no-reward, some previous studies have observed reduced conflict effects with reward in the Stroop but not the Simon task (see Table 1), suggesting a conflict-specific account for the Stroop and a performance-general account for the Simon task. To directly distinguish between these two accounts, all participants responded to the same target feature (color),^2^ but half completed the Simon task followed by the Stroop task, while the order was reversed for the other half. Reward prospect (reward vs. no-reward) was held constant within each block and alternated across blocks. In reward blocks, participants received a reward when their response was correct and below a certain individually adjusted threshold.

While all prior studies, except one, have employed trial-wise proactive reward manipulation, we chose to implement a block-wise manipulation, anticipating that this approach might yield more substantial main effects of rewards, as one might infer when comparing the previously reported main effects (see Table 1).^3^ A substantial reward effect is certainly advantageous for investigating potential modulations of conflict effects; if the reward manipulation generally more strongly affects processing, it might also more strongly modulate congruency effects if conflict-related processes are indeed sensitive to reward. For the same reason, we adopted an adaptive RT threshold instead of a fixed one. In this approach, the task-specific reward threshold remained constant within blocks but was adjusted on a block-wise basis according to the task-specific mean RT in correct no-reward trials from previous blocks (for a similar approach, see e.g., Kang et al., 2018; Soutschek et al., 2015). On a more theoretical level, we reasoned that choosing a block-wise design, rather than a trial-wise design, may allow participants to more effectively adjust their processing to the consistent reward (or no-reward) condition within blocks, as potential additional processes with a trial-wise manipulation, such as switching between cues (e.g., Jost et al., 2015), are avoided.

### Method

#### Participants

Forty^4^ participants (27 identifying as women and 13 identifying as men, 33 right-handed, *M*_age_ = 21.82 ± 2.7 years, range: 18–30) were recruited via advertisements on the campus of the University of Tübingen, social media, and internal departmental e-mail lists. They could receive course credits or money for their participation. Data of one participant were excluded due to low performance (<75% accuracy). All participants provided informed consent before testing, and they were tested in a single web-based online session lasting approximately 40 min.

#### Apparatus and stimuli

**Fig. 1:**
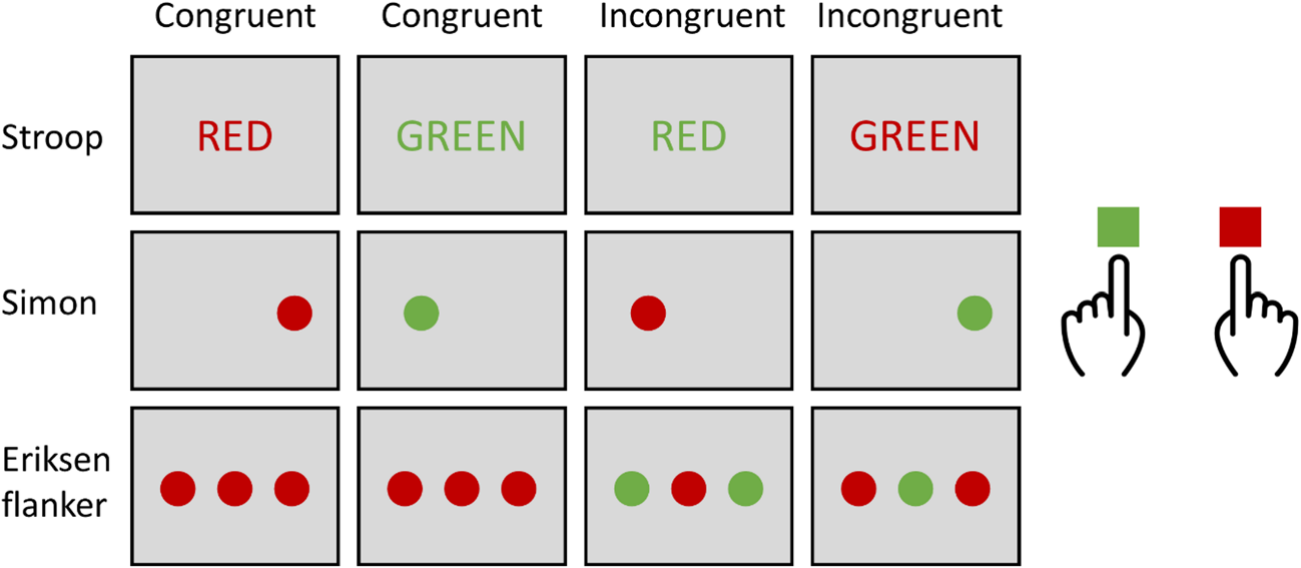
Schematic depiction of stimulus displays of the different conflict tasks (not to scale). (Color figure online)

Stimulus presentation and recording of responses were controlled by jsPsych (De Leeuw, 2015). All visual stimuli were presented on a grey background. A centrally positioned black plus sign (+) served as the fixation point. The target stimuli for the Simon task were red- and green-colored filled circles (see Fig. 1). The target stimuli for the Stroop task were the words “ROT” (German for “red”) and “GRÜN” (German for “green”) colored in red (RGB [139, 34, 34]) or green (RGB [34, 100, 34]). In Simon task blocks, the targets (colored circles) appeared either to the left or right of the center of the screen. In Stroop task blocks, the targets (colored words) appeared in the center of the screen. For each participant, red and green colors were randomly assigned to left- and right-hand responses. Responses were key presses with the left and right index fingers on the *Q* and *P* keys of a QWERTZ computer keyboard.

#### Procedure

Each participant was tested in 16 blocks with 48 trials per block (786 trials in total). In the first eight blocks, half of the participants performed the Stroop task and in the last eight blocks the Simon task, whereas this order was reversed for the other half of participants. Reward prospect (reward vs. no-reward) was held constant within a block and alternated across blocks. Half of the participants were tested with a reward block for the first block and the other half with no-reward for the first block. Each block consisted of 12 presentations of each of the four possible stimulus displays in the Simon task (i.e., two possible target colors × two locations) and the four stimulus possible displays in the Stroop task (i.e., two possible target ink colors × two distractor words). For reward blocks, participants were told that they would receive a reward for particularly fast and correct responses (+ 10 points). The currently accumulated points were displayed in advance of both reward and no-reward blocks. For no-reward blocks, participants were told that in this block, no-rewarding points could be gained.

**Fig. 2:**
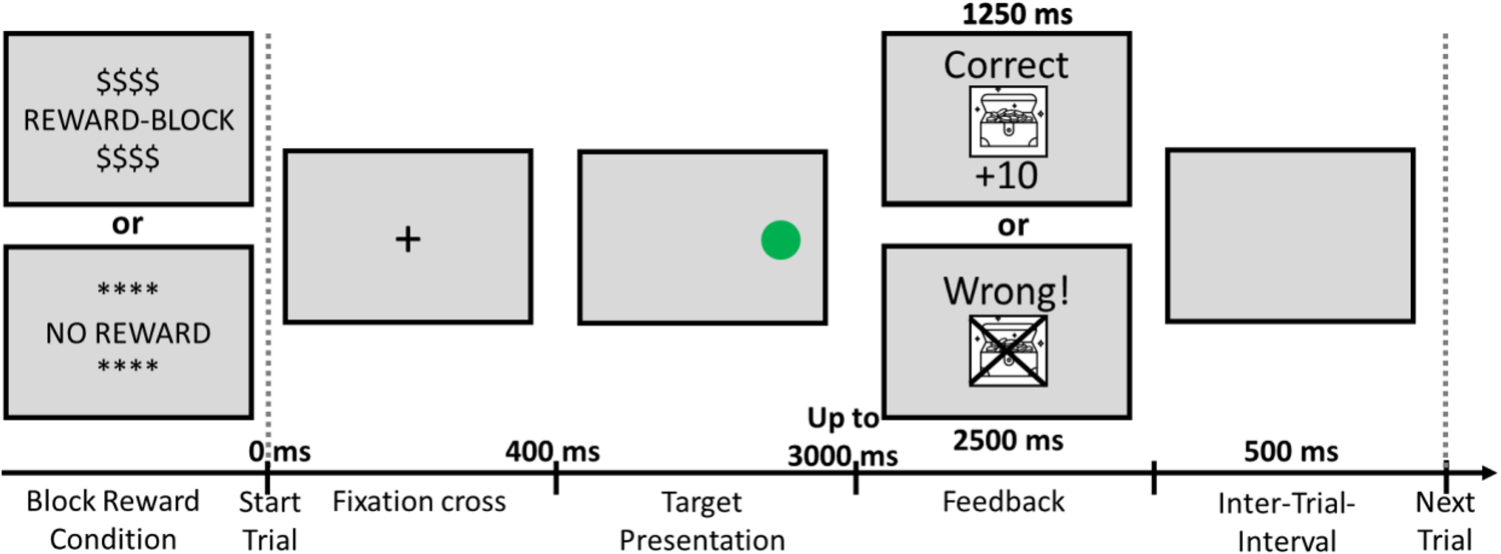
Schematic depiction of reward screen that appeared before each block as well as a possible trial sequence in Experiment 1

**Fig. 3:**
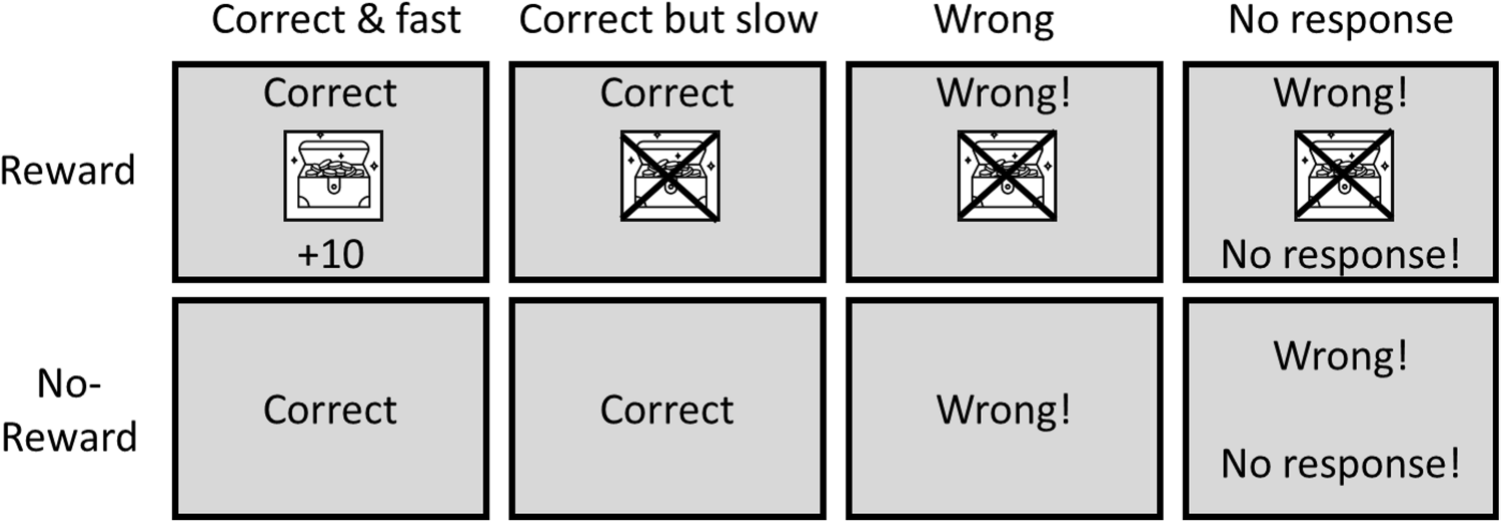
Feedback for reward and no-reward trials

Figure 2 illustrates a possible trial sequence. At the beginning of each trial, the fixation cross appeared on the screen for 400 ms. Following the offset of the fixation cross, a colored word was presented in the center of the screen (Stroop task), or the colored circle was presented to the left or right side of the screen (Simon task). The stimulus remained on the screen until participants responded, up to a maximum of 3 s.^5^ After each response, feedback was displayed for 1,250 ms when the response was correct and for 2,500 ms when it was wrong or no response was given within the overall RT deadline of 3 s. In reward blocks, feedback indicated whether the response was (1) “Richtig + 10 points!” (“Correct” in German); (2) “Richtig”; (3) “Falsch!” (“Wrong” in German); or (4) “Falsch! Keine Antwort!” (“Wrong! No Answer!” in German). For an overview, see Fig. 3. In addition, a treasure box was displayed if reward was obtained, whereas this treasure box was crossed out if no reward was obtained. The time limit for obtaining reward was adaptively calculated based on the average RTs of correct no-reward trials. In no-reward blocks, there was only written feedback indicating whether the response was (1) “Correct!”; (2) “Wrong”; or (3) “Too slow.” After each trial, there was an intertrial interval (ITI) of 500 ms. Breaks between blocks were self-paced, and the current points were displayed.

#### Data preparation and design^6^

For all analyses, the first two blocks of each task were considered practice (reward and no-reward) and therefore excluded. For both mean RT and mean PE, 2 × 2 × 2 analyses of variance (ANOVAs), with the three within-subject factors task (Simon vs. Stroop), reward prospect (reward vs. no-reward), and congruency (congruent vs. incongruent) were conducted. Furthermore, 2 × 2 within-subject ANOVAs with the factors reward and congruency were conducted separately for each conflict task.

In order to examine the conflict effects across the RT distribution, delta plots using five equally sized bins for the RT (and PE) data separately for each participant and within each condition were constructed, using the DMCfun R package (Mackenzie & Dudschig, 2021). To compare the time courses of the RT delta plots, we summarized the delta plot for each participant and each condition with a linear regression model predicting the delta in each bin from the mean RT in that bin. Following this, 2 × 2 ANOVAs, with the factors of conflict task and reward prospect, were calculated on the mean slopes. Furthermore, we calculated the congruency effects at a common value of RT, to control for time-varying fluctuations in congruency effects (cf. Mittelstädt & Miller, 2020). To do so, we used the regression model for each condition to compute the predicted congruency effect at each participant’s individual mean RT. Following this, 2 × 2 ANOVAs with the factors of conflict task and reward prospect were calculated on the predicted congruency effects.

### Results and discussion

#### Mean RT and mean ER

**Fig. 4:**
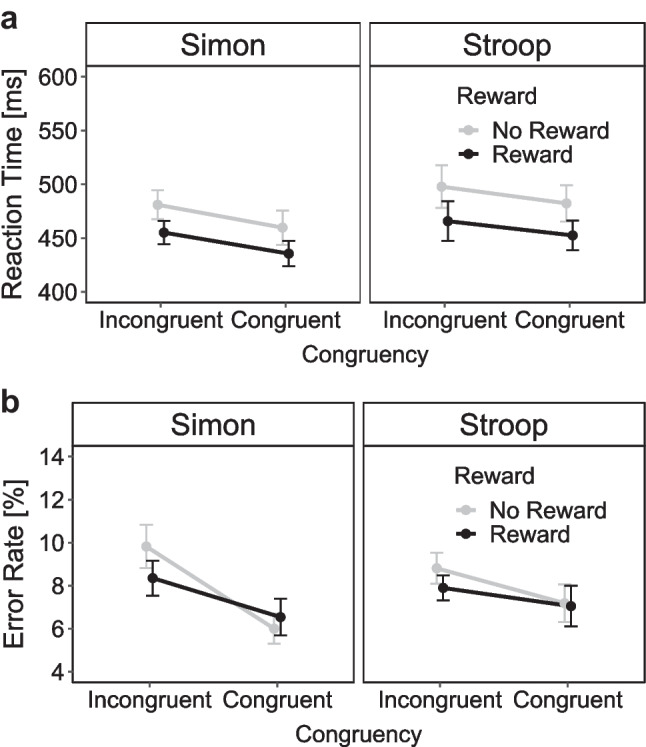
Mean reaction time in ms (**a**) and mean error rates in % (**b**) as a function of congruency (incongruent, congruent) and reward prospect (reward, no-reward) separately for the Simon and Stroop tasks in Experiment 1. *Note*. Error bars visualize standard errors of the mean

Figure 4 shows the mean RTs and mean ERs as a function of reward prospect and congruency separately for the two conflict tasks. The 2 × 2 × 2 ANOVA on mean RTs revealed a significant main effect of reward prospect indicating shorter RTs in rewarded compared with nonrewarded trials (Δ = 28 ms), *F*(1, 38) = 28.19, *p* < .001, η_p_^2^ = 0.43. A significant main effect of congruency reflected lower RTs in congruent trials compared with incongruent trials (Δ = 17 ms), *F*(1, 38) = 34.94, *p* < .001, η_p_^2^ = 0.48. The main effect of task was marginally significant, indicating higher RTs in the Stroop compared with the Simon task (Δ = 17 ms), *F*(1, 38) = 3.96, *p* = .054, η_p_^2^ = 0.09. No other effects were significant (all *p*s > .284, all η_p_^2^s < .03).

In the 2 × 2 ANOVA for the Simon task, there were significant main effects of congruency (Δ = 20 ms), *F*(1, 38) = 24.03, *p* < .001, η_p_^2^ = 0.39, and reward (Δ = 25 ms),* F*(1, 38) = 15.47, *p* < .001, η_p_^2^ = 0.29. The interaction was not significant (*p* = .815, η_p_^2^ < 0.01). Thus, there was no evidence that the size of the mean Simon effect differed between the reward (Δ = 19 ms) and no-reward (Δ = 22 ms) conditions. In the 2 × 2 ANOVA for the Stroop task, there were also significant main effects of congruency (Δ = 14 ms), *F*(1, 38) = 6.94, *p* < .012, η_p_^2^ = 0.15, and reward (Δ = 31 ms), *F*(1, 38) = 32.04, *p* < .001, η_p_^2^ = 0.46. Again, the interaction between congruency and reward was not significant (*p* = .689, η_p_^2^ < 0.01). Thus, there was also no evidence that the size of the mean Stroop effect differed between the reward (Δ = 14 ms) and no-reward (Δ = 16 ms) conditions.

The 2 × 2 × 2 ANOVA on mean ERs revealed only a significant main effect of congruency reflecting more errors in incongruent than congruent trials (Δ = 2.02%), *F*(1, 38) = 16.76, *p* < .001, η_p_^2^ = 0.31. No other effects were significant (all *p*s > .098, all η_p_^2^s < 0.07).

For the 2 × 2 ANOVA in the Simon task, there were only significantly more errors in incongruent than congruent trials (Δ = 2.81%), *F*(1, 38) = 8.97, *p* = .005, η_p_^2^ = 0.19, but neither a significant main effect of reward (*p* = .474, η_p_^2^ = 0.01) nor a significant interaction (*p* = .112, η_p_^2^ = 0.07). Thus, there was no evidence that the size of the Simon effect in error rates differed between the reward (Δ = 1.81%) and no-reward (Δ = 3.82%) conditions. For the 2 × 2 ANOVA in the Stroop task, there was also only a significant main effect of congruency (Δ = 1.23%), *F*(1, 38) = 4.19, *p* = .049, η_p_^2^ = 0.10 (with *p* = .396, η_p_^2^ = 0.02 for the main effect of reward and *p* = .459, η_p_^2^ = 0.01 for the interaction). Thus, there was no evidence that the size of the Stroop effect in error rates differed between the reward (Δ = 0.9%) and no-reward (Δ = 1.63%) conditions.

#### Distributional RT and ER

**Fig. 5:**
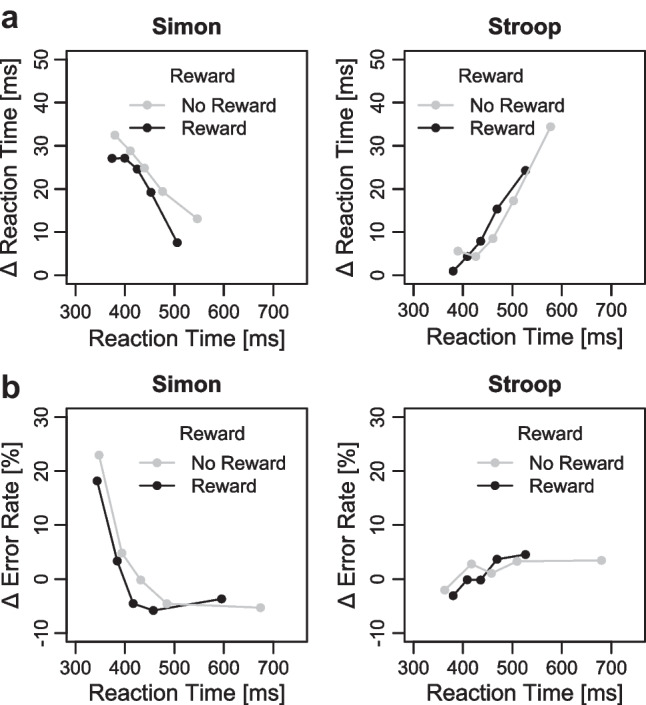
**a** Delta plots showing incongruent minus congruent differences in mean reaction time (RT) within each of five quantiles, plotted against the decile average RTs, separately for each reward condition and conflict task (Simon and Stroop) tasks in Experiment 1. **b** Delta plots showing incongruent minus congruent differences in mean error rates within each of five quantiles, plotted against the quantile averages separately for each reward condition and conflict task tasks in Experiment 1

Figure 5 shows the RT delta plots for the reward and no-reward conditions separately for the Simon and Stroop task. The 2 × 2 ANOVA on mean slopes of the RT delta plots revealed a main effect of task, *F*(1, 38) = 10.82, *p* = .002, η_p_^2^ = 0.22. As can be seen in Fig. 5a, the slopes were negative-going (−0.079) for the Simon task, but positive-going for the Stroop tasks (0.098). The main effect of reward (*p* = .339, η_p_^2^ = 0.02) and the interaction (*p* = .677, η_p_^2^ < 0.01) were not significant (with *p* = .323 and *p* = .741 for the pairwise comparisons of slopes within the Simon and Stroop tasks, respectively).

The 2 × 2 ANOVA on mean predicted congruency effects revealed no significant main effects of reward (*p* = .351, η_p_^2^ = 0.02) and task (*p* = .246, η_p_^2^ = 0.04), as well as no significant interaction (*p* = .318, η_p_^2^ = 0.03) (with *p* = .248 and *p* = .989 for the pairwise comparisons of predicted congruency effects within the Simon and Stroop task, respectively). Thus, there was no evidence for a reward specific modulation of congruency effects, even when controlling for the decrease (Simon) and increase (Stroop) of congruency effects over time. For completeness, we also computed delta plots in error rates (see Fig. 5b).

## Experiment 2

Experiment 1 revealed that task performance (as reflected in mean RT) was improved when anticipating rewards for both conflict tasks. Contrary to conflict-specific accounts, there was no evidence that reward prospect modulated the size of congruency effects in either conflict task. Experiment 2 was designed to investigate whether this result pattern will replicate when implementing a trial-based reward manipulation, as has been the case in most prior studies (cf. Table 1).

### Method

#### Participants

As preregistered, we collected the data of 40 participants (25 identifying as women and 15 identifying as men , 37 right-handed, *M*_age_ = 21.88 ± 3.31 years, range: 18–30), but data of three participants had to be excluded due to low performance (<75% accuracy).

#### Apparatus, stimuli, and procedure

**Fig. 6:**
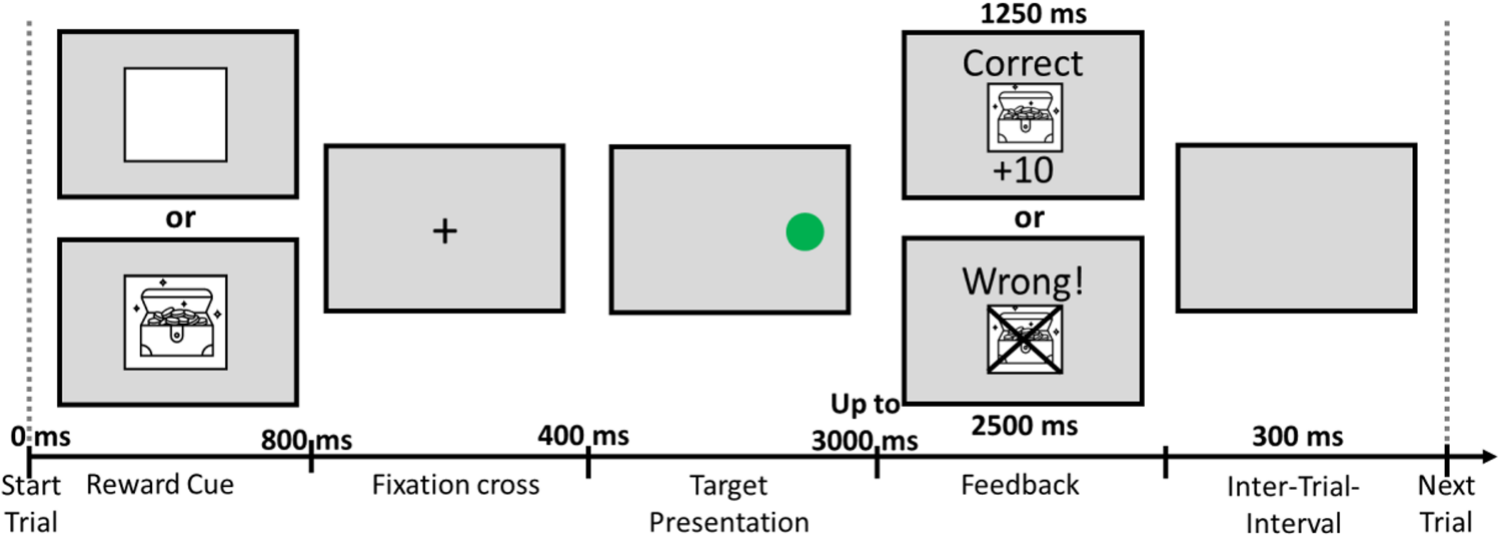
Schematic depiction of a possible trial sequence in Experiments 2 and 3

The methodological aspects were as in Experiment 1 except for the following changes. The reward prospect was manipulated trial-wise by presenting a cue for 800 ms indicating the reward condition before the fixation cross (see Fig. 6). Cues were either an open treasure chest (reward condition) or an empty white box (no-reward condition). Furthermore, the ITI was decreased to 300 ms, and the feedback duration was decreased to 750 ms for correct and 1,250 ms for incorrect answers (see Fig. 6). The number of blocks was also decreased to 14, to keep the number of used trials the same as in Experiment 1. Due to the trial-wise reward manipulation, only the first block of each task was considered practice and therefore excluded. As in Experiment 1, an individual RT reward threshold was employed, which remained constant within a block but was updated after each block based on the mean RT of no-reward trials.

### Results and discussion

#### Mean RT and mean ER

**Fig. 7:**
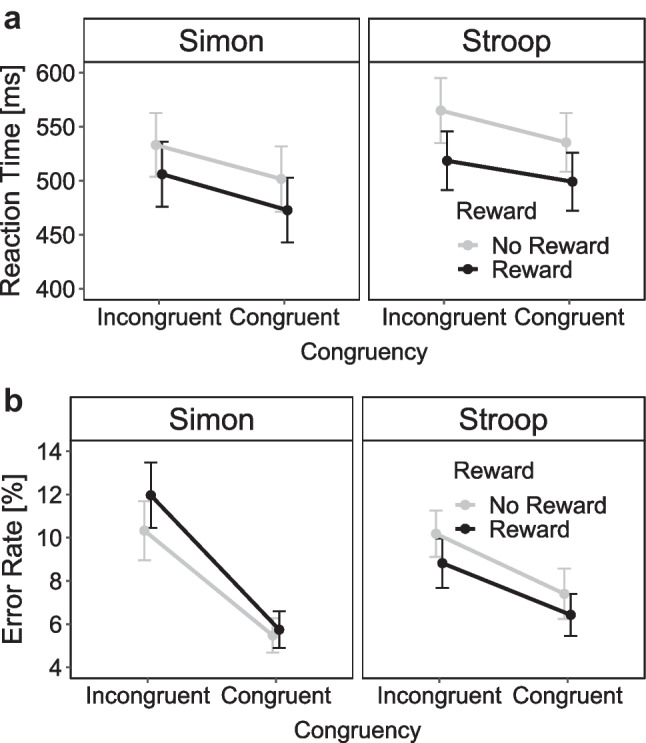
Mean reaction time in ms (**a**) and mean error rates in % (**b**) as a function of congruency (incongruent, congruent) and reward prospect (reward, no-reward) separately for the Simon and Stroop tasks in Experiment 2. *Note*. Error bars visualize standard errors of the mean

Figure 7 shows the mean RTs and mean ERs as a function of reward prospect and congruency separately for the two conflict tasks. The 2 × 2 × 2 ANOVA on mean RTs revealed a significant main effect of reward prospect indicating lower RTs in rewarded compared with nonrewarded trials (Δ = 35 ms), *F*(1, 36) = 27.00, *p* < .001, η_p_^2^ = 0.43. Furthermore, there were significant main effects of task, reflecting lower RTs in the Simon task compared with the Stroop task (Δ = 27 ms), *F*(1, 36) = 4.73, *p* = .036, η_p_^2^ = 0.12, and congruency, reflecting lower RTs in the congruent compared with the incongruent condition (Δ = 29 ms), *F*(1, 36) = 67.62, *p* < .001, η_p_^2^ = 0.65. Lastly, there was a significant interaction between task and reward, reflecting a larger reward effect in the Stroop than in the Simon task, *F*(1, 36) = 7.58, *p* = .009, η_p_^2^ = 0.17. No other interactions were significant (all *p*s > .151, all η_p_^2^s < .06).

In the 2 × 2 ANOVA for the Simon task, there were significant main effects of reward (Δ = 28 ms), *F*(1, 36) = 18.34, *p* < .001, η_p_^2^ = 0.34, and congruency (Δ = 32 ms), *F*(1, 36) = 74.80,* p* < .001, η_p_^2^ = 0.68 , but no interaction (*p* = .764, η_p_^2^ < 0.01). Thus, as for Experiment 1, there was no evidence that the size of the Simon effect differed between the reward (Δ = 34 ms) and no-reward (Δ = 31 ms) conditions. In the 2 × 2 ANOVA for the Stroop task, there were also significant main effects of reward (Δ = 41 ms), *F*(1, 36) = 29.35, *p* < .001, η_p_^2^ = 0.45, and congruency (Δ = 24 ms), *F*(1, 36) = 22.52, *p* < .001, η_p_^2^ = 0.38. Again, the interaction between congruency and reward was not significant (*p* = .081, η_p_^2^ = 0.08). Thus, there was again no evidence that the size of the Stroop effect differed between the reward (Δ = 20 ms) and no-reward (Δ = 29 ms) conditions.

The 2 × 2 × 2 ANOVA on mean ERs revealed a significant main effect of congruency reflecting more errors in incongruent than congruent trials (Δ = 4.06%), *F*(1, 36) = 28.69, *p* < .001, η_p_^2^ = 0.44, as well as a significant interaction between reward and task, *F*(1, 36) = 6.68, *p* = .014, η_p_^2^ = 0.16. This interaction indicated that participants made less errors in the reward than no-reward condition in the Stroop task, whereas the reverse was true for the Simon task. The interaction between reward and congruency was marginally significant, *F*(1, 36) = 3.89, *p* = .056, η_p_^2^ = 0.10, indicating that congruency effects in ERs were slightly larger in the no-reward than reward condition. No other effects were significant (all *p*s > .231, all η_p_^2^s < 0.04).

For the 2 × 2 ANOVA in the Simon task, there were only significantly more errors in incongruent than congruent trials (Δ = 5.53%), *F*(1, 36) = 18.33, *p* < .001, η_p_^2^ = 0.34. Neither a significant main effect of reward (*p* = .116, η_p_^2^ = 0.07) nor a significant interaction (*p* = .246, η_p_^2^ = 0.04) were observed. Thus, there was no evidence that the size of the Simon effect differed between the reward (Δ = 6.22%) and no-reward (Δ = 4.86%) conditions. For the 2 × 2 ANOVA in the Stroop task, there was also only a significant main effect of congruency (Δ = 2.59%), *F*(1, 36) = 11.26, *p* = .002, η_p_^2^ = 0.24. Neither the main effect of reward, *(p* = .089, η_p_^2^ = 0.08, nor the interaction (*p* = .679, η_p_^2^ < 0.01) was significant. Therefore, there was also no evidence for a reward-specific modulation of the Stroop effect (reward Δ = 2.40% and no-reward Δ = 2.78%).

#### Distributional RT and ER

**Fig. 8:**
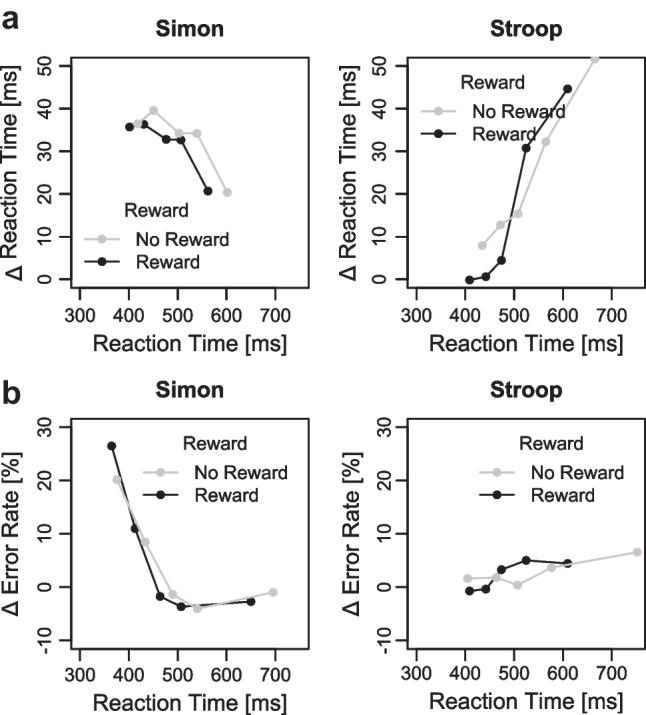
**a** Delta plots showing incongruent minus congruent differences in mean reaction time (RT) within each of five quantiles, plotted against the decile average RTs, separately for each reward condition and conflict task (Simon and Stroop) tasks in Experiment 2. **b** Delta plots showing incongruent minus congruent differences in mean error rates within each of five quantiles, plotted against the quantile averages separately for each reward condition and conflict task in Experiment 2

Figure 8a shows the RT delta plots for the reward and no-reward conditions separately for the Simon and Stroop tasks (see Fig. 8b for the delta plots in error rates). The 2 × 2 ANOVA on mean slopes revealed a main effect of task, *F*(1, 36) = 30.17, *p* < .001, η_p_^2^ = 0.46. The slopes were negative-going (−0.115) for the Simon task, but positive-going for the Stroop task (0.192). Neither the main effect of reward (*p* = .560, η_p_^2^ = 0.01) nor the interaction (*p* = .422, η_p_^2^ = 0.02) was significant (with *p* = .934 and *p* = .358 for the pairwise comparisons of slopes within the Simon and Stroop tasks, respectively).

The 2 × 2 ANOVA on mean predicted congruency effects reveled a significant main effect of task indicating smaller congruency effects in the Stroop (19 ms) compared with the Simon tasks (31 ms) when controlling for response speed, *F*(1, 36) = 5.16, *p* = .029, η_p_^2^ = 0.13. Neither the main effect of reward (*p* = .403, η_p_^2^= 0.02) nor the interaction (*p* = .231, η_p_^2^ = 0.04) was significant (with *p* = .211 and *p* = .997 for the pairwise comparisons of predicted congruency effects within the Simon and Stroop task, respectively). Thus, there was no evidence for conflict-specific accounts for prospective reward manipulations even when considering fluctuations of congruency effects across time.

## Experiment 3

Experiment 2 replicated the findings from Experiment 1, indicating an improvement in task performance (reflected in mean RTs) when reward was anticipated, but no-reward-specific modulation of congruency effects (neither in RTs nor error rates). Thus, both Experiments 1 and 2 provided no evidence for conflict-specific accounts and instead only offered evidence that reward generally improves performance. In Experiment 3, we again used a trial-wise reward manipulation but replaced the Stroop task with an Eriksen flanker task. Considering that a trial-wise manipulation is more commonly employed and, contrary to our initial reasoning, it appeared to us that the effects of reward, if any, were more substantial with a trial-wise than a block-wise manipulation (as observed in the main effects of reward between Experiments 1 and 2), we concluded that it would be sufficient to exclusively test the flanker effect using this manipulation.

As mentioned in the introduction, one previous study has reported reduced flanker effects when participants anticipated performance-contingent reward (e.g., Yamaguchi & Nishimura, 2019), but it is possible that this finding is due to differences in cue presentation between reward conditions (i.e., cue in the reward, but no cue in the no-reward condition). We decided to conduct another test of the influence of reward prospect in the Simon task, as the delta plots of the reward condition were descriptively below the delta plots of the no-reward condition, as predicted by conflict-specific accounts. Considering that potential reward-specific modulations of congruency effects were smaller than expected, we decided to increase the sample size from 40 to 60 participants.

### Method

#### Participants

As preregistered, we collected the data of 60 participants (46 identifying as women and 14 identifying as men, 51 right-handed, *M*_age_ = 21.48 ± 3.97 years, range: 18–46), but data of two participants had to be excluded due to poor performance (<75% accuracy).

#### Apparatus, stimuli, and procedure

The methodological aspects were as in Experiment 2, except that we replaced the Stroop task with an Eriksen flanker task (see Fig. 6).

### Results and discussion

#### Mean RT and mean ER

**Fig. 9:**
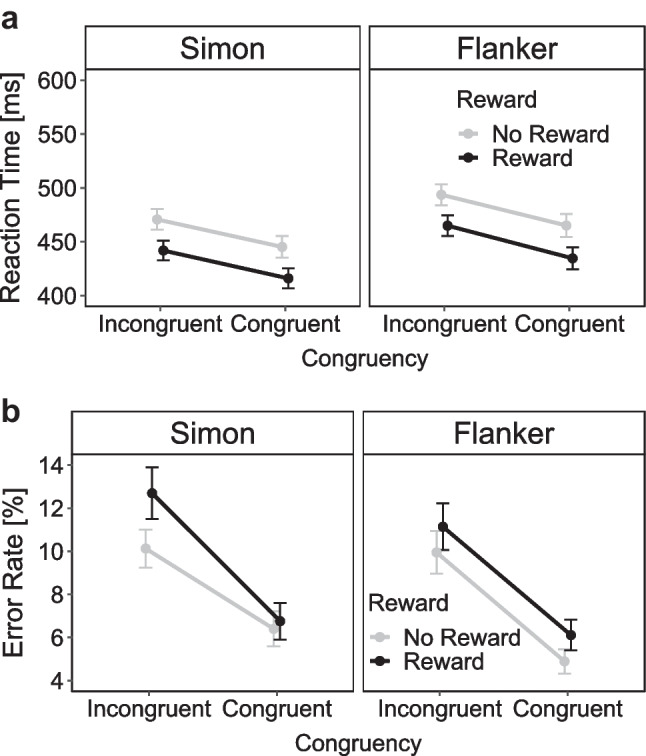
Mean reaction time in ms (**a**) and mean error rates in % (**b**) as a function of congruency (incongruent, congruent) and reward prospect (reward, no-reward) separately for the Simon and Eriksen flanker tasks in Experiment 3. *Note.* Error bars visualize standard errors of the mean

Figure 9 depicts the mean RTs and mean ERs as a function of reward prospect and congruency separately for the two conflict tasks. The 2 × 2 × 2 ANOVA on mean RTs revealed a significant main effect of reward prospect due to lower RTs in the reward compared with no-reward condition (Δ = 30 ms), *F*(1, 57) = 43.12, *p* < .001, η_p_^2^ = 0.43. The main effect of task indicated lower RTs in the Simon compared with the Eriksen task (Δ = 22 ms), *F*(1, 57) = 17.30, *p* < .001, η_p_^2^ = 0.23. The main effect of congruency reflected lower RTs in the incongruent compared with the congruent condition (Δ = 28 ms), *F*(1, 57) = 173.47, *p* < .001, η_p_^2^ = 0.75. There were no significant interactions (all *p*s > .352, all η_p_^2^s < .02).

For the 2 × 2 ANOVA for the Simon task, there were significant main effects of reward (Δ = 29 ms), *F*(1, 57) = 28.46, *p* < .001, η_p_^2^ = 0.54, and congruency (26 ms), *F*(1, 57) = 28.46, *p* < .001, η_p_^2^ = 0.33, but no interaction *F*(1, 57) = 0.01, *p* = .917, η_p_^2^ < 0.01. As for the previous two experiments, there was no evidence that the size of the Simon effect differed between the reward (Δ = 26 ms) and no-reward (Δ = 26 ms) conditions. The 2 × 2 ANOVA for the Eriksen flanker task also revealed main effects of reward (Δ = 29 ms), *F*(1, 57) = 46.23, *p* < .001, η_p_^2^ = 0.45, and congruency (Δ = 29 ms), *F*(1, 57) = 124.80, *p* < .001, η_p_^2^ = 0.69, but no interaction *F*(1, 57) = 0.29,* p* = .595, η_p_^2^ = 0.00. Thus, there was no evidence for a reward-specific modulation of flanker effects (reward Δ = 30 ms and no-reward Δ = 29 ms).

The 2 × 2 × 2 ANOVA on mean ERs revealed a significant main effect of congruency, reflecting more errors in incongruent than congruent trials (Δ = 4.94%), *F*(1, 57) = 64.92, *p* < .001, η_p_^2^ = 0.53. Interestingly, a significant main effect of reward indicated slightly more errors in the reward compared with no-reward conditions (Δ = −1.33%), *F*(1, 57) = 14.34, *p* < .001, η_p_^2^ = 0.20. The main effect of task (*p* = .131, η_p_^2^ = 0.04) and the interaction between task and congruency (*p* = .841, η_p_^2^ < 0.01) were not significant. However, both the interaction between reward and congruency (*p* = .074, η_p_^2^ = 0.05), as well as the three-way interaction (*p* = .064, η_p_^2^ = 0.06) were marginally significant.

For the 2 × 2 ANOVA in the Simon task, there were significantly more errors in incongruent than congruent trials (Δ = 4.83%), *F*(1, 57) = 24.94, *p* < .001, η_p_^2^ = 0.30. Additionally, there was a main effect of reward with more errors in the reward compared with the no-reward condition (Δ = 1.45%), *F*(1, 57) = 8.96 *p* = .004, η_p_^2^ = 0.14. The interaction between reward and congruency was also significant, *F*(1, 57) = 7.03 *p* = .010, η_p_^2^ = 0.11. Surprisingly, the size of the Simon effect in error rates was larger in the reward (Δ = 5.94%) than no-reward (Δ = 3.73%) condition. For the 2x2 ANOVA in the Eriksen flanker task, there were only significant main effects of reward (Δ = −1.21%), *F*(1, 57) = 7.42, *p* = .009, η_p_^2^ = 0.12, and congruency (Δ = 5.00%), *F*(1, 57) = 71.35, *p* < .001, η_p_^2^ = 0.56 , but no significant interaction *(p* = .976, η_p_^2^ < 0.01) . Thus, there was no evidence that the size of the error-based flanker effect differed between the reward (Δ = 5.03%) and no-reward (Δ = 5.05%) conditions.

#### Distributional RT and ER

**Fig. 10:**
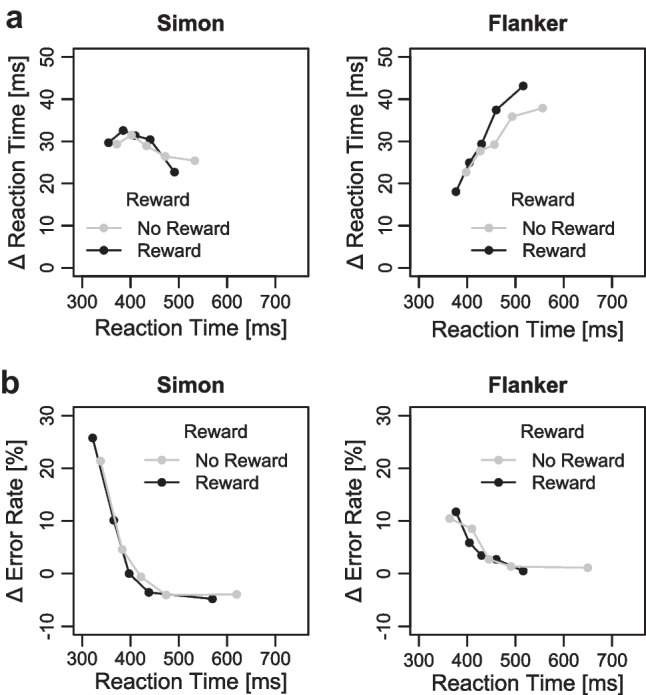
**a** Delta plots showing incongruent minus congruent differences in mean reaction time (RT) within each of five quantiles, plotted against the decile average RTs, separately for each reward condition and conflict task (Simon and Stroop) in Experiment 3. **b** Delta plots showing incongruent minus congruent differences in mean error rates within each of five quantiles, plotted against the quantile averages separately for each reward condition and conflict task in Experiment 3

Figure 10a shows the RT delta plots for the reward and no-reward condition separately for the Simon and Eriksen flanker task (see Fig. 10b for the delta plots in error rates). The 2 × 2 ANOVA on mean slopes revealed a main effect of tasks, *F*(1, 57) = 32.88, *p* < .001, η_p_^2^ = 0.37. While the slopes were once again negative-going (−0.038) for the Simon task, they were positive-going for the Eriksen flanker task (0.186). There was no main effect of reward (*p* = .298, η_p_^2^ = 0.02), but an interaction between task and reward *F*(1, 57) = 4.11, *p* = .047, η_p_^2^ = 0.07. The delta plot slopes in the Eriksen flanker task were more strongly increasing in the reward compared with no-reward condition (with *p* = .036 for the pairwise comparisons), whereas there was no difference in delta plot slopes for the Simon task (*p* = .465).

The 2 × 2 ANOVA on mean predicted congruency effects revealed no significant main effects of task or reward (all *p*s > .347, η_p_^2^ < 0.02) and no significant interaction (*p* = .263, η_p_^2^ = 0.02, with *p* = .211 and *p* = .969 for the pairwise comparisons of predicted congruency effects within the Simon and Eriksen Flanker tasks, respectively). Thus, these analyses provide no evidence for a reward-specific modulation of congruency effects when controlling for time-varying fluctuations of distractor-based activation.

## General discussion

In the present study, we investigated the impact of performance-contingent reward prospect on task performance across three conflict tasks. Specifically, we investigated whether reward anticipation leads to overall performance improvements or can also influence the effect of distracting information in Simon and Stroop tasks (Experiment 1 and 2, respectively) and in Simon and Eriksen flanker tasks (Experiment 3). In Experiment 1, we manipulated reward prospect between blocks, while in Experiment 2 and 3 we manipulated it trial-wise. Across all experiments, task performance generally improved with reward compared with no-reward prospect, but there was no conclusive evidence that the mean conflict effects were modulated by reward prospect, as would be predicted by conflict-specific behavioral accounts.

Contrary to some previous studies, but in line with one other (Bundt et al., 2016), the present study does not provide evidence for conflict-specific accounts in terms of reduced mean Stroop, Eriksen flanker, and Simon RT effects when reward was available for good performance. While this does not rule out the possibility that reward prospect manipulations may modulate these effects under some circumstances, our results suggest that if these interactive effects do exist, they are likely to be small and may require larger sample sizes and/or may depend on specific methodological aspects that we can currently only speculate about.^7^ For example, reduced flanker effects with performance-contingent reward compared with no-reward prospect may be due to the presentation of a reward cue only on rewarded trials (Yamaguchi & Nishimura, 2019).^8^ Another example is that the reduced Stroop effects with reward compared with no-reward prospect may be limited to picture-word Stroop-like tasks (e.g., Padmala & Pessoa, 2011; Soutschek et al., 2015). Furthermore, it may also be relevant to consider how performance-contingent rewards are computed. For instance, while the use of an adaptive RT threshold, as in our current study, has been shown to elicit modulations in conflict effects within the context of studies employing both proactive (e.g., Soutschek et al., 2015) and reactive reward manipulations (cf. Chen et al., 2021; Mittelstädt et al., 2023b), maintaining a globally constant reward rate of 50% might potentially discourage participants from engaging in conflict-sensitive control processes (for more discussion, cf. Prével et al., 2021). Finally, it also appears plausible that conflict-sensitive control induced by a reward cue may only (or more strongly) come into effect when participants receive additional information about the upcoming trial type (i.e., incongruent vs. congruent; cf. Chiew & Braver, 2016). In any case, given that we observed generally similar results across different conflict tasks while keeping other aspects alike, the theoretically more interesting possibility of distractor-specific effects of proactive reward influences, as observed for reactive reward influences (Mittelstädt et al., 2022), seems unlikely.

The present study also contributes to the literature by investigating whether evidence for conflict-specific accounts can be found when examining the RT distribution via delta plots. This is particularly important because, across all experiments, the conflict effects varied with response speed, with smaller conflict effects for faster than slower responses in the Stroop and Eriksen flanker tasks, and the reverse being true for the Simon task. These differences in the slope of the delta plots are usually considered to reflect time-varying distractor-based activation and examining only mean RTs can make it difficult to understand whether similar or different conflict effects across conditions are confounded by time-dependent distractor processing (e.g., Mittelstädt & Miller, 2020; Mittelstädt et al., 2022). For example, a similar mean Simon effect in the two reward conditions could have been observed because, on the one hand, the Simon effect increases with faster response times under reward due to generally higher absolute distractor activation, but on the other hand, the Simon effect decreases under reward because distractor suppression is stronger, which should decrease the relative contribution of distractor activation. However, despite controlling for response speed, we found no evidence for reward-specific modulation on conflict effects in any of the three experiments. Moreover, the similar shapes of task-specific delta plots across reward conditions suggest that reward prospect does also not affect the timing of conflict-related control (e.g., speed of distractor suppression) as has been often observed with other factors (e.g., reactive reward manipulation in the Eriksen flanker task, cf. Mittelstädt et al., 2022; proportion congruency manipulation in the Simon task, cf. Hübner and Töbel 2019).

The specific causes underlying the impact of reward prospects when making decisions under conflict are not entirely clear. One explanation could be that the possibility of reward only influences aspects of target processing that are not involved in conflict resolution. Considering that the pattern remained largely consistent across various distractors that induce perceptual-related (e.g., Eriksen) and motor-related (e.g., Simon) informational conflicts (e.g., Hommel, 2011), as well as higher-level task conflicts (e.g., Stroop; cf. Goldfarb & Henik, 2007), this might suggest that either very early sensory or late motor processes are improved in anticipation of performance-contingent rewards. However, it is also possible that the similar conflict effects observed in behavior across reward and no-reward conditions are due to a reward-specific enhancement of target processing at the conflict resolution stage, which is accompanied by increased distractor sensitivity, as participants might generally be in a heightened state of arousal in the reward condition (cf. Lloyd & Nieuwenhuis, 2024). Thus, a more complex possibility is that the reward manipulation could also strengthen target processing under reward at the stage where conflict occurs, which would normally lead to reduced conflict effects. However, this effect may be countered by increased distractor-based activation under reward, resulting in similar conflict effects across reward and no-reward conditions in the observed behavior.

Furthermore, it is also useful to consider that our primary focus thus far has been on reward-based performance improvements in RTs and the corresponding potential modulation of RT conflict effects. Similar to some prior studies (e.g., Bundt et al., 2016; Soutschek et al., 2015; Yamaguchi & Nishimura, 2019), we did not observe any significant reward effects on error rates in the present study, except for Experiment 3: Surprisingly, there were significantly more errors in the reward condition compared with the no-reward condition in this experiment. This observation might suggest that participants, especially in this experiment, additionally attempted to lower their response threshold in order to meet the reward-RT threshold, even if it resulted in an increase in error rates. This adjustment could have also contributed to the significant increase in the Simon effect observed in error rates in the reward condition compared with the no-reward condition in this experiment: As Simon effects are typically more pronounced for fast responses in error rates, the act of speeding up responses by lowering the response threshold in the reward condition could result in significant Simon effects in error rates for fast responses, precisely as we observed in the error-based delta plots (see Fig. 10b). While this pattern was only observed for very fast responses and was not replicated in either Experiment 1 or 2 (see also Footnote 6), it seems still worthwhile to consider in future studies that a performance-contingent proactive reward cue may not consistently be used to enhance all facets of performance. Moreover, we hope that the consistent empirical RT pattern in terms of performance-general accounts across different conflict tasks and more fine-grained behavioral measures (delta plots) in the present study will serve as a solid foundation for a more precise distinction between the underlying factors that give rise to the observed behavior.

## Footnotes

1 In these studies, it is usually assumed that reward modulates learning-related cognitive control processes (e.g., strengthening target–response or distractor–response associations) that operate reactively during a trial, as participants do not know in advance of each trial whether features with or without rewards are presented.

2 Similar to previous studies investigating the influence of reward in conflict tasks (e.g., Padmala & Pessoa, 2011; Mittelstädt et al., 2023b; Soutschek et al., 2015), we only focused on visual conflict tasks with two manual responses. Thus, it remains to be seen to what extent the current findings generalize to conflict tasks with auditory stimuli, vocal responses, and/or more complex response mappings (e.g., four manual responses).

3 Specifically, as shown in Table 1, the two studies with blockwise reward manipulation had considerably larger main effects of reward on RTs (>64 ms) than the other studies (<41 ms), and in both of these studies, congruency effects were modulated by the reward manipulation.

4 As can be seen in our preregistration, we have not provided a sample-size justification there. The sample size was somewhat arbitrarily determined, but we considered both practical constraints (e.g., participants availability) and empirical constraints (e.g., effect size in previous studies, greater variability in an online setting). With the chosen sample size, we would achieve over 80% power to detect reward-specific modulations of congruency effects of at least η_p_^2^ = .18 at a significance level of α = 0.05. We considered this to be a reasonable minimum effect size of interest, as previous studies typically reported larger interaction effects when they were present (e.g., η_p_^2^ = .20 in Soutschek et al., 2015).

5 In general, we opted for a relatively long RT deadline of 3 s to ensure that we cover the effects of the reward manipulation across a wide range of RTs via the delta plot. Also, in a prior study using a Simon–Stroop task with a reactive reward manipulation (Mittelstädt et al., 2023b), the effects were quite clear in both the mean and distributional RT and ER patterns even without any RT deadline. Note also that the reward-specific RT deadline was considerably lower for all participants in each experiment, the results in each experiment remained similar when additional RT cutoffs were applied (e.g., excluding all trials slower than 2 s or 1 s), and the delta plots provide little evidence, if any, that the reward manipulation differentially interacts with the congruency effects observed with different response speeds. Of course, it is in principle possible that with a lower overall (reward unspecific) RT deadline, participants might make more errors, and as a result, this might increase the chances of observing potential effects of reward in error rates. However, on the other hand, this might also make it more challenging to observe strong reward effects in RTs (i.e., if participants are already required to respond very fast in the no-reward condition, the reward-RT threshold may be quite similar).

6 We would like to note that we have not mentioned any data exclusion criteria in our preregistration. However, virtually similar results were observed when including the first two blocks or when additionally applying RT cutoffs (e.g., 2 s or 1 s). Moreover, as mentioned in our preregistration, we have also attempted to fit the data of all experiments to the diffusion model of conflict (DMC) using the R package DMCfun (Mackenzie & Dudschig, 2021). Unfortunately, we were unable to reasonably fit the DMC to the data, and the reliability of the estimated parameters was relatively poor. Therefore, we have decided not to report any DMC modeling in the present study.

7 In additional exploratory (not preregistered) analyses, we combined the data from Experiments 1 and 2 for the Stroop task (*N* = 76) and the data from all experiments for the Simon task (*N* = 134). We then conducted separate ANOVAs for each task with these larger sample sizes, examining both mean RT and mean error rates. However, for both measures, the interactions were not significant in either the Stroop or Simon task (all *p*s > .127, η_p_^2^ < .04). Moreover, we also explored the possibility that a potential effect of reward on conflict effects would only emerge when considering the data where the respective conflict task was applied in the first half. Our rationale was that participants might be more inclined to engage conflict-specific control processes in the initial half of each experiment based on the reward cue, as they could become fatigued and/or increasingly perceive themselves as unable to enhance their performance due to the adaptive threshold (cf. Prével et al., 2021). However, even in these analyses conducted on the relevant data (aggregated across experiments for Simon and Stroop), the interactions for both mean RT and mean error rates did not reach statistical significance (all *p*s > .150, η_p_^2^ < .04).

8 Note that this issue was already recognized by the authors of this previous study and addressed in follow-up experiments in which they implemented a performance-*independent* reward prospect manipulation. Moreover, it is worth noting that their study primarily focused on investigating the influence of reward on the congruency sequence effect.

## Declarations

### Open practice statement

Preregistrations and raw data of all experiments are available via the Open Science Framework (OSF; https://osf.io/dbeq2/). Materials for the experiments reported here are available from the authors upon request.
